# Effects of visualization of successful revascularization on chest pain and quality of life in chronic coronary syndrome: study protocol for the multi-center, randomized, controlled PLA-pCi-EBO-pilot-trial

**DOI:** 10.1186/s13063-020-04710-7

**Published:** 2020-10-08

**Authors:** Michael Wester, Franziska Koll, Florian Zeman, Astrid Dempfle, Michael Koller, Norbert Frey, Lars S. Maier, Samuel Sossalla

**Affiliations:** 1 University Heart Centre Regensburg, Department of Internal Medicine II, University Hospital Regensburg, Regensburg, Germany; 2 Centre for Clinical Studies, University Medical Centre, Regensburg, Germany; 3 Institute of Medical Informatics and Statistics, University Medical Centre Schleswig-Holstein, Campus Kiel, Kiel, Germany; 4 Department of Internal Medicine III, University of Kiel, Kiel, Germany

**Keywords:** Chronic coronary syndrome, Stable coronary artery disease, Placebo effect, Angina pectoris

## Abstract

**Background:**

Stable coronary artery disease (CAD), recently termed chronic coronary syndrome (CCS), is a highly prevalent disease. Current treatment strategies often include a relevant placebo effect. The hypothesis is that visual angiographic demonstration of the coronary arteries before and after successful percutaneous coronary intervention (PCI) by itself reduces the symptom burden of stable CAD/CCS.

**Design and methods:**

The PLA-pCi-EBO-pilot-trial is a prospective, multi-center, randomized, controlled investigator-initiated pilot trial to study the effect of visual demonstration of successful PCI on quality of life (QoL) and angina pectoris (AP) in patients with symptomatic stable CAD/CCS. All patients with stable CAD/CCS and successful PCI will be screened. One hundred forty four patients with a frequency of AP ≥ 2/week will be randomized 1:1 stratified for AP frequency > 1/day. The control group will receive the common written procedural report on the procedure. Patients in the intervention group will additionally be given a printout picture of their coronary angiogram both before and after PCI. Primary endpoints are change in the Seattle Angina Questionnaire (SAQ)-derived QoL score 1 and 6 months after PCI. Secondary endpoints are changes in other SAQ-derived scores and dyspnea (NYHA score) 1 and 6 months after PCI.

**Discussion:**

The PLA-pCi-EBO-pilot-trial evaluates the effect of visual angiographic result demonstration on disease symptoms and QoL in patients with stable CAD/CCS on top of PCI. A positive outcome of our study would encourage the routine use of angiographic picture demonstration and has thus the potential to change daily routine in the catheterization laboratory.

**Trial registration:**

German Clinical Trials Register DRKS00017524. Registered on 5 July 2019

## Administrative information

The order of the items has been modified to group similar items (see http://www.equator-network.org/reporting-guidelines/spirit-2013-statement-defining-standard-protocol-items-for-clinical-trials/).

**Table:** 

Title {1}	Effects of visualization of successful revascularization on chest pain and quality of life in chronic coronary syndrome: study protocol for the multi-center, randomized, controlled PLA-pCi-EBO-pilot-trial
Trial registration {2a and 2b}.	German Clinical Trials Register DRKS00017524.
Protocol version {3}	Original (2019-June-26)
Funding {4}	University Hospital Regensburg, Doktor Robert Pfleger Stiftung Bamberg Germany
Author details {5a}	University Heart Centre Regensburg, Department of Internal Medicine II, University Hospital Regensburg, Regensburg, Germany: Michael Wester, Franziska Koll, Lars S. Maier, Samuel Sossalla University Medical Centre, Centre for Clinical Studies, Regensburg, Germany: Florian Zeman, Michael Koller Institute of Medical Informatics and Statistics, University Medical Centre Schleswig-Holstein, Campus Kiel: Astrid Dempfle Department of Internal Medicine III, University of Kiel, Kiel, Germany: Norbert Frey
Name and contact information for the trial sponsor {5b}	Universitätsklinikum Regensburg Franz-Josef-Strauß-Allee 11 93053 Regensburg Germany Phone: 09419447210 URL: http://ukr.de
Role of sponsor {5c}	This funding source had no role in the design of this study and will not have any role during its execution, analyses, interpretation of the data, or decision to submit results.

## Introduction

### Background and rationale {6a}

Among patients between 40 and 79 years of age, almost 10% are affected by coronary artery disease (CAD), and the prevalence is rising with growing age [1]. CAD significantly reduces life expectancy by promoting cardiac infarction and development of ischemic heart disease [2]. Patients are often suffering from symptoms such as angina pectoris (AP) and dyspnea, potentially resulting in dramatically reduced quality of life (QoL). Medical and interventional treatment aims to restore optimal coronary blood flow, modify cardiovascular risk factors, reduce cardiac remodeling, treat possible heart failure, and implement secondary prevention. To highlight the chronic nature and dynamic processes of CAD, the latest update of the ESC guidelines changed the term stable CAD to chronic coronary syndrome (CCS) [2, 3]. This term rightly represents an altered and more accurate concept of CAD. For the purpose of avoiding confusion, especially since almost all studies cited in this article have been conducted using the previous nomenclature, we deliberately simplify the differences between the two terms and use stable CAD and CCS as synonyms.

Despite optimal interventional and medical treatment, the symptoms of CAD are often poorly controlled. The COURAGE and FAME2 trials found that appr. 20–30% of patients still suffered from persistent AP and dyspnea on exertion after 1 year of optimal treatment [4, 5]. It is striking that in studies examining antianginal therapies, a large symptom relief in the placebo group can be observed. This is evident, for example, in studies evaluating ranolazine [6] or ivabradine [7] which reported a reduction of the AP frequency of up to 40% in the placebo study arm. The ORBITA trial was published as a seminal work investigating the effects of PCI in patients with stable angina in a randomized, sham-controlled fashion. However, there was no overall difference between groups for exercise capacity testing, angina, or quality of life [8]. Taken together, these results indicate that a relevant part of the symptomatic improvement after medical or interventional treatment might be attributed to the placebo effect and context factors. To further enhance the efficacy of interventions, profound knowledge and understanding of context factors are essential.

The concept of placebo medicine is complex and no clear definition exists [9]. Generally speaking, placebo constitutes the substitution of the active ingredient of a therapeutic intervention through an inactive ingredient in a manner that is indistinguishable for the recipient. Even for a drug with a known mechanism of action, the drug’s pharmacological effect cannot be separated from the psychological effects of its administration and the accompanying physiological consequences [10]. Growing evidence exists for possible mechanisms of the placebo effect. Placebo interventions activate the endogenous opioid system [for review see [11]] and the dopaminergic reward circuit which is crucial for reinforcement, learning, and decision-making [for review see [11]]. Placebos also affect the autonomous nervous system for instance by altering the duration of gastric contractions [10]. The placebo effect is strongly influenced by patients’ expectations [12], learning [12], emotions [13], and personality traits such as optimism [14], empathy [12], and reward responsiveness [14].

Communication forms an essential part of the patient-doctor relationship and is key to ensure patients’ understanding of their disease and treatment and thus propagate therapy adherence. Improved communication is an important context factor often implicated as a huge contributor to the placebo effect [15]. Visualization is a good and effective means to facilitate communication and risk evaluation between patients and the physician. The VIPVIZA trial highlights the importance of the visualization of diagnostic findings to the patient [16].

### Objectives {7}

Given the still limited treatment options for stable CAD and the often significantly reduced quality of life, we hypothesized that the visualization of the severe coronary stenosis before and the angiographic result after successful coronary stent implantation will reduce angina pectoris and thereby improve quality of life.

### Trial design {8}

The PLA-pCi-EBO-pilot-trial is a prospective, multi-center, randomized, controlled investigator-initiated pilot trial to study the effect of visual demonstration of successful coronary intervention on quality of life and angina pectoris in patients with symptomatic stable coronary artery disease. The trial has been approved by the Ethical Committee of the University of Regensburg (19-1261-101). The German Clinical Trials Register registration number is DRKS00017524.

## Methods: participants, interventions, and outcomes

### Study setting {9}

Patients will be recruited in Germany from different clinical settings including a university hospital, a community hospital, and a cardiological clinic.

### Eligibility criteria {10}

Patients with clinical symptoms of stable coronary artery disease such as typical pain, chest discomfort (e.g., pressure, tightness, squeezing, burning), or atypical angina together with dyspnea undergoing cardiac catheterization and stent implantation will be screened for inclusion into the study. The decision for PCI and the choice of implemented technique, stents, balloons, etc. is made by the treating cardiologist based on current guidelines [2] and best clinical practice.

Inclusion criteria are:

1. Females or males age ≥ 18 years
2. Symptomatic CAD
3. CCS score for angina pectoris ≥ 2
4. Angina pectoris frequency ≥ 2/week
5. Implantation of ≥ 1 coronary artery stent
6. German speaking
7. Signed informed consent

Abbreviations: CAD, coronary artery disease; CCS, Canadian Cardiovascular Society

Exclusion criteria are:

1. Cardiac EF ≤ 35%
2. Severe pulmonary disease
3. Impaired vision
4. Impaired hearing
5. Dementia
6. Treatment with opioids
7. High-grade cardiac valvular disease
8. Hb ≤ 7 mg/dL
9. Participation in another trial of an investigational drug or device

Abbreviations: EF, ejection fraction; Hb, hemoglobin

### Who will take informed consent? {26a}

Written informed consent will be taken after a trained participating consultant has explained the study’s aims, conduct, and procedures to the patients and answered possible questions.

### Additional consent provisions for collection and use of participant data and biological specimens {26b}

The study does not include ancillary studies or the collection of biological specimens.

### Interventions

#### Explanation for the choice of comparators {6b}

Our approach of influencing AP frequency by the simple means of visual demonstration of the angiographic result of PCI is very pragmatic. As no clear recommendation exists, current practice is that some interventionalists demonstrate the interventional result to the patient during the PCI procedure. This will be noted but does not constitute an exclusion criterion because the aim of this study is to compare the explicit visual demonstration of successful PCI to the current clinical practice. The patients in the control group receive only the usual doctor’s note at hospital discharge without any pictorial representation of the treated stenosis.

#### Intervention description {11a}

**Fig. 1:**
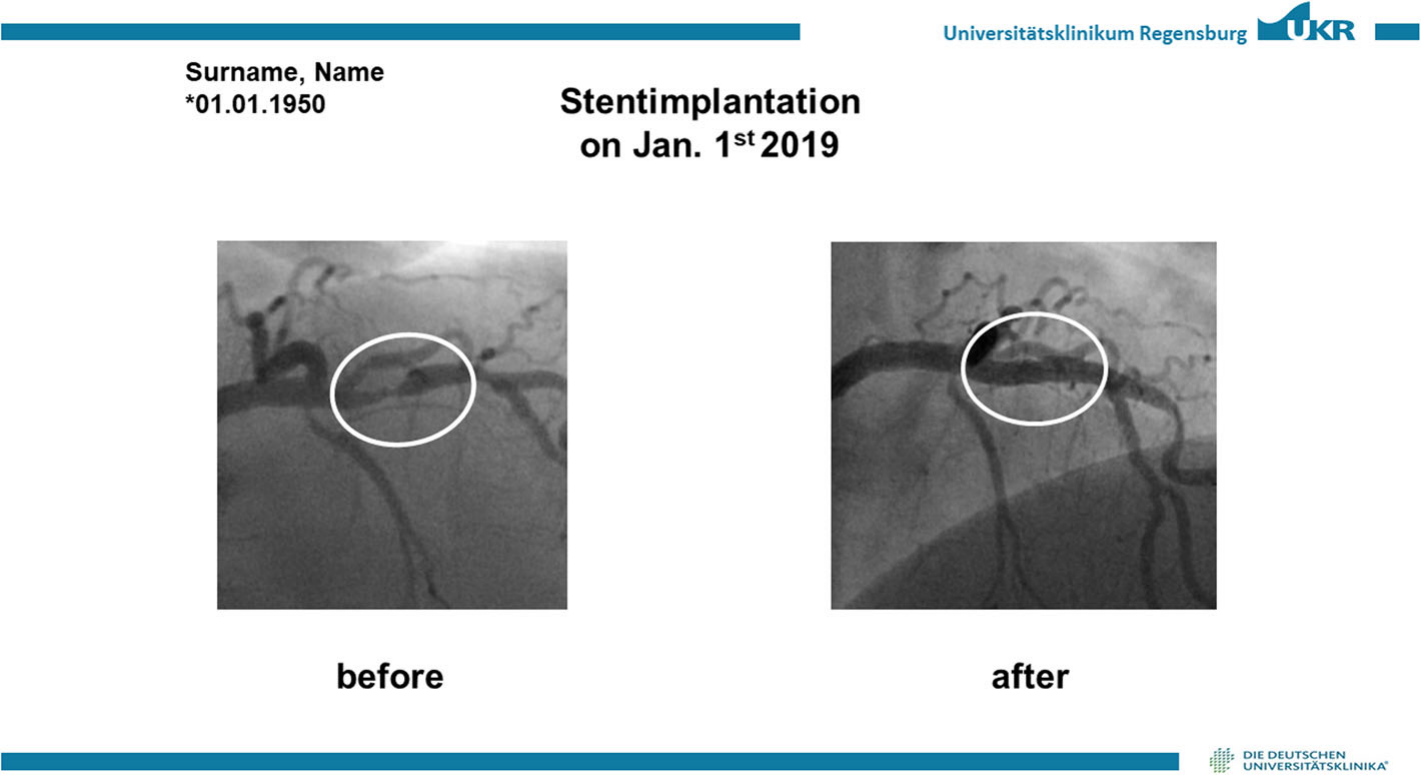
Anonymized patient printout of the successful PCI. On the left a coronary angiography of the stenosis is shown and on the right after successful coronary intervention. For better understanding, the stenosis is highlighted by a white circle

The patients in the control arm will only receive the written report on the procedure as is current common clinical practice. Patients in the intervention arm will be additionally given a printout of their coronary status (picture angiogram) both before and after PCI to emphasize the outcome of the successful therapy (see Fig. 1). The prints and coronary intervention will be briefly explained to the patient by a qualified physician.

#### Criteria for discontinuing or modifying allocated interventions {11b}

As the intervention consists of demonstrating a picture of the angiogram to the patients after successful PCI, there are no serious adverse reactions caused by the intervention to be expected.

#### Strategies to improve adherence to interventions {11c}

The follow-up interviews include the question, if and how often patients in the intervention group have looked at the angiogram picture after hospital discharge. This constitutes necessary information to estimate the relevance and meaning of the picture for the patients. The patients are not encouraged to look at the picture either at the baseline visit or the follow-ups in order to protect this information from being influenced by the investigators’ behavior.

#### Relevant concomitant care permitted or prohibited during the trial {11d}

Routine practice, medication, and clinical check-ups after PCI or possible participation in cardiac rehabilitation programs are not affected by the study protocol.

#### Provisions for post-trial care {30}

Given the harmless nature and singularity of the intervention, no post-trial care is planned. Patients that have been randomized to the control group can request a printout of their angiogram after completion of the study.

### Outcomes {12}

Symptoms such as AP according to the Canadian Cardiovascular Society score and dyspnea according to the NYHA scale will be assessed. The German version of the revised Life Orientation Test (LOT-R) [17] and the German version of the Seattle Angina Questionnaire (SAQ, CV outcomes Inc.) [18] will be filled out by the patient before randomization.

The primary endpoints are:

- Change from baseline in the SAQ-derived quality of life score 1 and 6 months after PCI

The secondary endpoints are:

- Change from baseline in the other SAQ-derived scores (physical limitation, angina stability, angina frequency, treatment satisfaction, disease perception)
- Change from baseline in the total SAQ score 1 and 6 months after PCI
- Change from baseline in dyspnea (NYHA score) 1 and 6 months after PCI

#### Participant timeline {13}

**Fig. 2:**
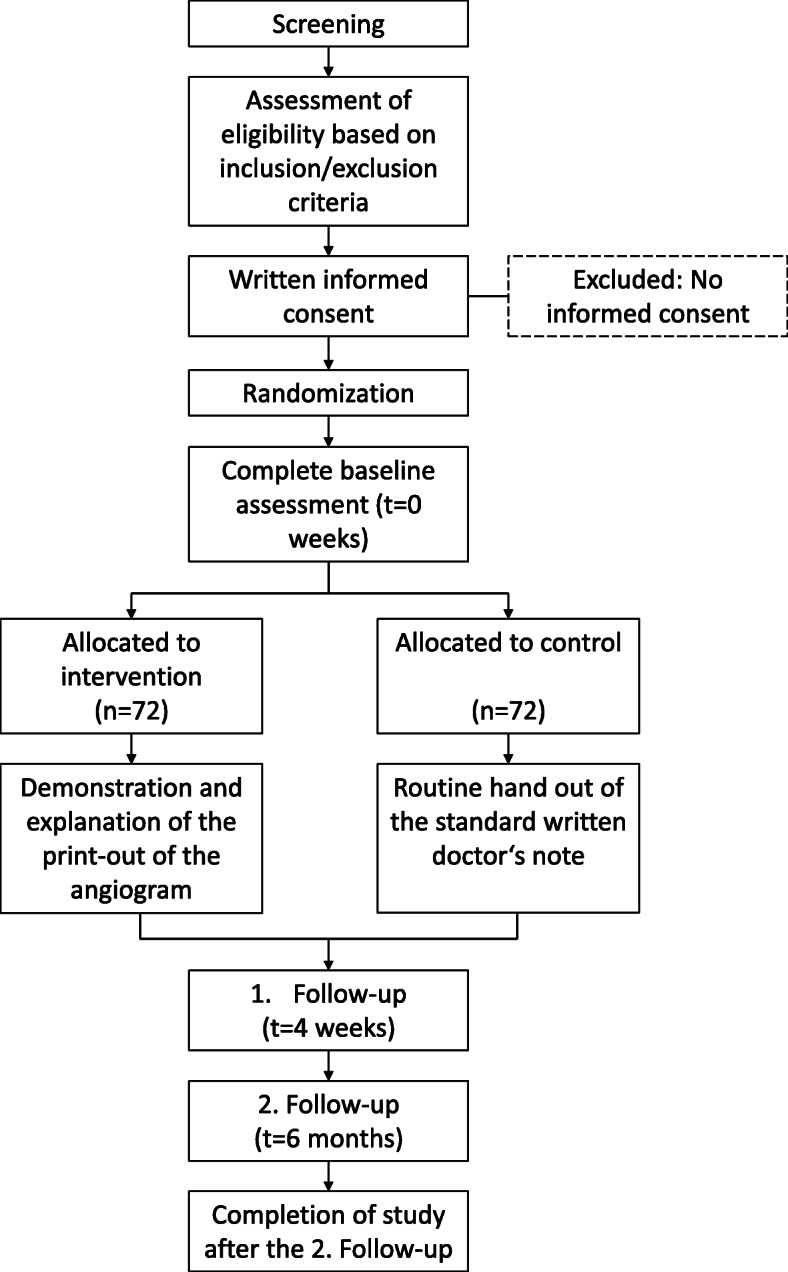
Participant timeline

**Fig. 3:**
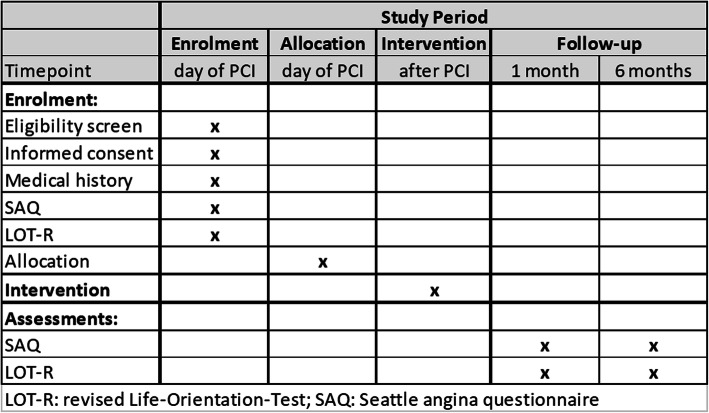
SPIRIT figure

Patients with clinical symptoms of stable CAD such as typical pain, chest discomfort (e.g., pressure, tightness, squeezing, burning), or atypical angina together with dyspnea undergoing cardiac catheterization and stent implantation will be screened for inclusion into the study. One hundred thirty-four patients who fulfill the inclusion and exclusion criteria will be randomized stratified by angina frequency (daily vs not daily) in a 1:1 ratio to receive either only the standard written report or an additional printout of the images acquired during catheterization of both the severe coronary stenosis before and after stent implantation. Baseline scores of dyspnea (NYHA score) and SAQ are measured during the initial hospital visit. After 4 weeks and 6 months, the SAQ will be repeated and the NYHA score reassessed in a telephone interview (see Figs. 2 and 3).

#### Sample size {14}

The study is designed and powered as a pilot trial to allow for precise parameter estimation for a subsequent confirmatory study. There is sparse comparable data to calculate effect size estimations for the kind of intervention employed in our study. However, clinical observation suggests that our pragmatic approach of influencing AP frequency by the simple means of visual demonstration of the angiographic result of PCI has a strong effect on the patients. The ISCHEMIA trial revealed relevant symptomatic improvement in patients with more severe AP [19]. Placebo- and sham-controlled trials have shown that large parts of the treatment effect of conservative and interventional therapeutic strategies for AP can be ascribed to context factors such as the one employed in this pilot study. Thus, we think it is reasonable and realistic to expect a difference of 10 points with a standard deviation of 20 between the control and interventional groups regarding the quality of life scale of the SAQ (effect size of *d* = 0.5 (medium effect)). This difference would also be highly clinically relevant. To show an effect size of *d* = 0.5 with a power of 80% (*β* = 0.2) at a two-tailed significance level of *α* = 0.05, *n* = 64 patients need to be included in each group. With an estimated drop-out rate of 10%, a total of *n*_total_ = 144 patients need to be recruited.

#### Recruitment {15}

More than 2000 PCIs are performed at the study centers each year. Patient lists are screened at the end of each day for probable participants (i.e., successful PCI and chronic coronary syndrome) and eligibility criteria are checked. It is estimated that approximately 200 (i.e., 10% of the total) of the patients present with chronic coronary syndrome.

### Assignment of interventions: allocation

#### Sequence generation {16a}

Patients who fulfill the inclusion and exclusion criteria will be randomized in a 1:1 ratio stratified for daily or not daily AP episodes by a computer-generated random number list.

#### Concealment mechanism {16b}

The allocation sequence is hidden in sequentially numbered, opaque, sealed envelopes.

#### Implementation {16c}

The allocation sequence is provided by the Centre for Clinical Studies, Regensburg, Germany. After written informed consent and evaluation of baseline characteristics, the envelopes are opened by the study doctor, and the patient is allocated to the intervention or control group.

### Assignment of interventions: blinding

#### Who will be blinded {17a}

Due to the nature of the study treatment, the study is open labeled, i.e., neither the patient nor the study physician are blinded. Nevertheless, patients are not explicitly informed about the exact difference between the study and the control group as they are told that different forms of communication are being tested. The follow-up telephone interviews are performed by a blinded study doctor.

#### Procedure for unblinding if needed {17b}

In this open-label trial, no unblinding needs to be performed.

### Data collection and management

#### Plans for assessment and collection of outcomes {18a}

The primary and secondary endpoints of our trial are mainly based on the scales of the SAQ. These include evaluation of QoL, physical limitation, angina stability, angina frequency, treatment satisfaction, and disease perception. The SAQ is the most widely used and extensively validated patient-reported measure of outcome for AP. Originally derived from a mostly male cohort [18], it has since also been validated for women [20–23]. The SAQ scores were shown to correlate with physiological metrics of coronary dysfunction and to be of prognostic value in patients with obstructive CAD [4, 21, 22].

Dyspnea is not part of the definition of angina pectoris; however, a substantial part of patients with CAD present with dyspnea as the dominant symptom together with atypical chest pain [24]. We additionally investigate changes of the NYHA classification at the time of both follow-ups. This classification has been widely used to assess dyspnea especially in the setting of heart disease and is part of the evaluation recommended in the current guidelines [2].

Data collection also includes clinical baseline parameters such as age, sex, complaints, severity of CAD, left ventricular ejection fraction, heart frequency, blood pressure, and medication.

#### Plans to promote participant retention and complete follow-up {18b}

The participants will be contacted via telephone for the 1- and 6-month follow-up. The participants will not receive monetary compensation.

#### Data management {19}

Data entry is performed both on the case report form and an electronic database. To ensure optimal quality control regarding GCP-compliant data management, monitoring, and biometry, the Centre for Clinical Studies at the University Hospital Regensburg is part of the trial.

#### Confidentiality {27}

All study-related information will be stored securely at the study site. All participant information will be stored in locked file cabinets in areas with limited access. All reports, data collection, process, and administrative forms will be identified by a coded ID (identification) number only to maintain participant confidentiality. All records that contain names or other personal identifiers, such as locator forms and informed consent forms, will be stored separately from study records identified by code number. All local databases will be secured with password-protected access systems. Forms, lists, logbooks, appointment books, and any other listings that link participant ID numbers to other identifying information will be stored in a separate, locked file in an area with limited access.

#### Plans for collection, laboratory evaluation, and storage of biological specimens for genetic or molecular analysis in this trial/future use {33}

No biological specimens are collected for this study.

### Statistical methods

#### Statistical methods for primary and secondary outcomes {20a}

The primary and all secondary endpoints will be analyzed by using an analysis of covariance (ANCOVA) with treatment group and sex as fixed factors and baseline measurement of the dependent variable, number of implanted stents, and baseline of Canadian Cardiovascular Society score as covariates. All analyses will be performed by using the intention-to-treat population. Patients with missing data in one of the endpoints will be excluded from the respective analysis.

#### Interim analyses {21b}

Interim analyses are not planned.

#### Methods for additional analyses (e.g., subgroup analyses) {20b}

Further group analyses will be stratified by frequency of AP ≤ 2/week and severity of CAD (> 1 vessel affected). As dyspnea is a common symptom of CAD which is not used as a criterion for the classification of typical angina pectoris [2], we will also analyze the pre-specified subgroup of people presenting with dominant dyspnea as an equivalent of AP.

#### Methods in analysis to handle protocol non-adherence and any statistical methods to handle missing data {20c}

Data will be analyzed using the intention-to-treat principle. Crossover in our study would mean that patients in the control group have access to the angiogram of their PCI. However, these data are not routinely sent to other hospitals, primary care physicians, or care givers. Therefore, we expect the crossover rate to be very low.

#### Plans to give access to the full protocol, participant-level data, and statistical code {31c}

The datasets generated and analyzed during this study are available from the corresponding author on reasonable request.

### Oversight and monitoring

#### Composition of the coordinating center and trial steering committee {5d}

The coordinating center is the University Heart Centre Regensburg, Department of Internal Medicine II, University Hospital Regensburg, Regensburg, Germany. In each participating center, a lead investigator (cardiologist) will be identified, to be responsible for identification, recruitment, data collection, and completion of CRFs, along with follow-up of study patients and adherence to study protocol. To ensure optimal quality control regarding GCP-compliant data management, monitoring, and biometry, the Centre for Clinical Studies at the University Hospital Regensburg is part of the trial.

#### Composition of the data monitoring committee, its role, and reporting structure {21a}

The data monitoring committee consists of the key persons for conducting this trial (Samuel Sossalla, Michael Wester, and Franziska Koll) and will maintain monthly meetings to discuss the progress and possible harms of this trial. Stopping rules include slow recruitment and life-threatening events, such as heart failure.

#### Adverse event reporting and harms {22}

This trial does not include the application of medicinal products or medical devices. Thus, no formal adverse event reporting is implemented. Nevertheless, as described above in **{21a}**, the progress of the trial conduct including potential harms is regularly discussed within the trial team.

#### Frequency and plans for auditing trial conduct {23}

Independent monitoring is provided by the Centre for Clinical Studies at the University Hospital Regensburg according to a pre-specified monitoring plan.

#### Plans for communicating important protocol amendments to relevant parties (e.g., trial participants, ethical committees) {25}

Any modifications to the protocol which may impact the conduct of the study and potential benefit of the patient or may affect patient safety, including changes of study objectives, study design, patient population, sample sizes, study procedures, or significant administrative aspects, will require a formal amendment to the protocol and approval by the local Ethics Committee prior to implementation and will be brought to the attention of the health authorities in accordance with local regulations.

### Dissemination plans {31a}

The papers presenting the results of this trial, especially baseline characteristics, and data of both follow-ups will be reviewed and approved by Samuel Sossalla, Michael Wester, and Franziska Koll.

## Discussion

Symptoms of stable CAD are currently often insufficiently controlled and have been shown to be highly prone to placebo interventions and modulation of context factors. Directly influencing these factors probably offers a means to reduce disease burden and improve QoL in this patient group. The PLA-pCi-EBO-pilot-trial examines the effects of visual demonstration of successful PCI in patients with stable CAD/CCS.

Daily clinical experience suggests that the integration of the patient into the decision-making process and appropriate, yet sufficiently detailed explanation of procedures and their results markedly improve therapy satisfaction and outcomes. An easy technique by which to achieve this is visual demonstration of test and therapeutic results, e.g., graphs of blood test results, x-ray images, etc. This assumption is derived from clinical experience and scarce scientific data exists to support this notion. Visualization of cardiovascular risk using risk scores, color-coded charts, graphs, and icons have been shown to alter patients’ behavior and reduce cardiovascular risk factors [25, 26]. These assumptions of the power of visualization were highlighted and confirmed by the VIPVIZA trial. In this Swedish trial, a stylized picture of patients’ carotid status, plaques, and stenosis as detected by ultrasound were shown and handed out to the patients. This led to a significant reduction of cardiovascular risk (Framingham risk score, European systematic coronary risk evaluation SCORE) in the 1-year follow-up [16]. The EISNER trial took a similar approach by assessing the effects of coronary artery calcium scanning on coronary risk factors. Patients that were randomized to coronary artery calcium scanning had a lower Framingham risk score compared to the control group in the 4-year follow-up [27]. These studies illustrate that visualization may constitute a potent means to alter patients’ cardiovascular risk, probably by altering lifestyle decision and improving adherence to medical treatment.

Comparable results were also found in oncology. In a randomized controlled trial (RCT) on patients with breast cancer, the intervention arm involved a graphic quality of life profile directed towards the patients’ treating physicians, whereas the control arm did not receive this visual aid. Half a year after the start of treatment, patients in the intervention arm reported better quality of life than patients in the control arm [28]. A recent RCT on patients with colorectal cancer confirmed this finding [29].

The PLA-pCi-EBO-pilot-trial aims to utilize these and other observations and translate them to improve outcomes of patients with stable CAD. This is especially relevant as this pathology represents a disease that is highly prevalent, limiting, and often difficult to treat. More than one third of patients in the COURAGE trial were still suffering from chest pain and dyspnea on exertion despite optimal treatment after 1 year [4]. Many trials investigating strategies to reduce disease burden have demonstrated a high susceptibility of AP for sham medication or interventions. Trials studying medical interventions in order to treat symptomatic patients frequently report highly relevant reductions in AP frequency in the placebo group [6, 7]. The ORBITA trial, a seminal work investigating the role and effects of PCI in patients with stable angina in a randomized, sham-controlled fashion, showed no difference between placebo and intervention groups for exercise testing, angina, or quality of life [8]. This study indicates that a relevant amount of the improvement after PCI might be attributed to the placebo effect and context factors. However, it has to be noted that the ORBITA trial only investigated one vessel disease in a highly selected, rather oligosymptomatic patient group and that there is a large debate on how these results should be interpreted and what they mean for clinical practice [30]. Major criticisms included that the follow-up time was rather short and a large number of patients crossed over from the placebo group to the PCI group.

Finally, the primary endpoint of this study consists of QoL in patients with CCS which is also considered to be related to the degree and the location of coronary stenosis. However, one major aim of PCI in CCS is symptomatic relief irrespective of specific lesion criteria [2]. The effect of PCI can be divided into measures caused by relief of a pathological mismatch which is certainly dependent on the initial problem, i.e., lesion specifications. However, as in any other medical intervention, this effect is accompanied by changes in disease perception and central pain processing (see the “Introduction” section). Therefore, we do not believe that the effect of our intervention—or placebo in general—is limited to specific lesions but rather relies on modulating the individual response of the participant, e.g., by improving disease perception, confidence in the treatment, and by reducing anxiety.

A picture conveys the information about the patient’s vascular status much more readily than explaining it in theory. Thus, demonstration of the results of the angiography is a possibility to positively alter context factors. Many practitioners already employ this method directly in the catheterization laboratory to explain the complex procedure. However, patients are often overwhelmed when this information is presented to them directly after the intervention. Routine visual demonstration after the PCI is not practiced yet. Our study does not exclude patients who have already been demonstrated the angiographic result in the catheterization laboratory as our aim is to evaluate the effect on top of this brief visual demonstration. However, it will be registered in our study if the physician has already shown the patient angiographic pictures during the intervention. Additionally, patients receive a printout of their angiography so that they have a reminder of the successful intervention after hospital discharge. The follow-ups include evaluation if and how often the patient has looked at the printouts after hospital discharge. As education might influence patients’ susceptibility for our intervention and understanding of the PCI procedure, this trait will also be registered. It is reasonable to assume that not every patient will profit from visual demonstration. Our study will include evaluation of a simple score to a priori estimate the success of our intervention.

Every medical encounter is characterized by a highly specific patient-clinician relationship. Although it is difficult to assess the precise effect of this relationship on medical outcomes, a meta-analysis of randomized controlled trials examining different communicational interventions found a small yet significant effect. The effect size was comparable to the reduction of myocardial infarction rates when using aspirin [15]. The placebo effect is moderated by stable personality traits such as optimism [14], empathy [12], and reward responsiveness [14], as well as transient psychological factors such as expectations [12], learning [12], or emotions [13]. Improvement of communication can positively influence these transient factors. Our study aims to elicit the placebo effect by modifying the context factors of PCI. We employ the German version of the revised Life Orientation Test [17] to assess the impact of dispositional optimism on the effect of visual demonstration.

Dyspnea is not part of the definition of angina pectoris; however, a substantial part of patients with CAD present with dyspnea as the dominant symptom together with atypical chest pain [24]. The latest update of the ESC guideline on stable CAD/CCS acknowledges this fact and highlights dyspnea as an important symptom of CAD [2, 3]. Clinical experience suggests that patients presenting with dyspnea as the main symptom are less susceptible to conventional medical or interventional treatment of CAD. This group therefore shows an increased need for additional treatment options and might especially benefit from alterations of context factors as are examined in our study design. Therefore, we additionally investigate changes of the NYHA classification at the time of both follow-ups. The time frame of the telephone interviews was chosen to assess for short-term effects after 1 month when the memory of the visual demonstration is supposedly still vivid. However, patients often do not recommence their normal daily activities directly after hospital discharge and therefore may not have had sufficient time to experience the full effects of PCI or of the visual demonstration. The 6-month interval was chosen to account for this and to measure possible lifestyle-induced changes in outcome.

The PLA-pCi-EBO-pilot-trial is designed as a pilot study to allow for precise parameter estimation for a subsequent confirmatory study. The determination of the study group size was based on both clinical and methodological considerations as well as on empirical facts since there are no clear reference points in the literature for this kind of investigation. Nevertheless, our considerations are informed enough to qualify this project as a pilot study that may set the stage for a large-scale trial with a definitive clinical impact. Clearly, obtaining the hypothesized effect justifies replication in a large-scale multi-center trial. Even if the hypothesized effect falls short of the conventional criteria of statistical significance, we may gain valuable information for designing a more definitive trial.

Quite generally, we believe that the subject of this study is highly relevant as it examines a simple, cost- and time-efficient, virtually side-effect-free tool to alleviate disease burden in the large and growing group of patients suffering from symptomatic stable CAD/CCS which has the potential to change the daily routine in the catheterization laboratory.

## Trial status

The trial is currently in the recruitment phase. The first participant was randomly assigned on 29 April 2019. At the time of the manuscript submission, we had recruited one third of the calculated example size. Expected completion for the recruitment phase is October 2020 and for study completion April 2021. The final protocol version is version “final form 1.0, 3 July 2019”.

## Abbreviations

AP: Angina pectoris
CAD: Coronary artery disease
CCS: Chronic coronary syndrome
EF: Ejection fraction
ESC: European Society of Cardiology
GCP: Good clinical practice
Hb: Hemoglobin
ID: Identification
LOT-R: Revised Life Orientation Test
NYHA: New York Heart Association
PCI: Percutaneous coronary intervention
QoL: Quality of life
RCT: Randomized controlled trial
SAQ: Seattle Angina Questionnaire
